# Effectiveness of Kaolinite with and Without Polyaluminum Chloride (PAC) in Removing Toxic *Alexandrium minutum*

**DOI:** 10.3390/toxins17080395

**Published:** 2025-08-06

**Authors:** Cherono Sheilah Kwambai, Houda Ennaceri, Alan J. Lymbery, Damian W. Laird, Jeff Cosgrove, Navid Reza Moheimani

**Affiliations:** 1 Algae Innovation Hub, Murdoch University, Perth, WA 6150, Australia; sheilah.kwambai@murdoch.edu.au (C.S.K.); houda.ennaceri@murdoch.edu.au (H.E.); a.lymbery@murdoch.edu.au (A.J.L.); d.laird@murdoch.edu.au (D.W.L.); jeff.cosgrove@dwer.wa.gov.au (J.C.); 2 Centre for Sustainable Aquatic Ecosystems, Harry Butler Institute, Murdoch University, Perth, WA 6150, Australia; 3 Department of Biodiversity, Conservation and Attractions, 17 Dick Perry Ave, Kensington, WA 6151, Australia

**Keywords:** harmful algal blooms, removal rates, zeta potential, cost estimate, *Alexandrium* spp., modified clays, kaolinite, seawater

## Abstract

*Alexandrium* spp. blooms and paralytic shellfish poisoning pose serious economic threats to coastal communities and aquaculture. This study evaluated the removal efficiency of two *Alexandrium minutum* strains using natural kaolinite clay (KNAC) and kaolinite with polyaluminum chloride (KPAC) at three concentrations (0.1, 0.25, and 0.3 g L^−1^), two pH levels (7 and 8), and two cell densities (1.0 and 2.0 × 10^7^ cells L^−1^) in seawater. PAC significantly enhanced removal, achieving up to 100% efficiency within two hours. Zeta potential analysis showed that PAC imparted positive surface charges to the clay, promoting electrostatic interactions with negatively charged algal cells and enhancing flocculation through Van der Waals attractions. In addition, the study conducted a cost estimate analysis and found that treating one hectare at 0.1 g L^−1^ would cost approximately USD 31.75. The low KPAC application rate also suggests minimal environmental impact on benthic habitats.

## 1. Introduction

The harmful algal bloom (HAB)-forming dinoflagellate microalgae *Alexandrium* spp. have caused socioenvironmental disruption around the globe [1,2,3,4]. Blooms of *Alexandrium* are known for their toxicogenic diversity, producing three distinct families of toxins: spirolides, goniodomins, and saxitoxins [2]. Saxitoxins are the most significant of these paralytic shellfish toxins (PSTs) and the main cause of paralytic shellfish poisoning in humans [5], a severe, and occasionally fatal, illness [6,7,8,9]. In addition to public health effects, PSTs can pose threats to marine organisms in the food web, including shellfish, planktivorous fish, and seabirds [2,10,11], impacting aquatic ecosystems and economic activities [12]. *Alexandrium* spp. are also known to produce allelochemicals that act against the wider plankton community [13,14,15].

**Figure 1:**
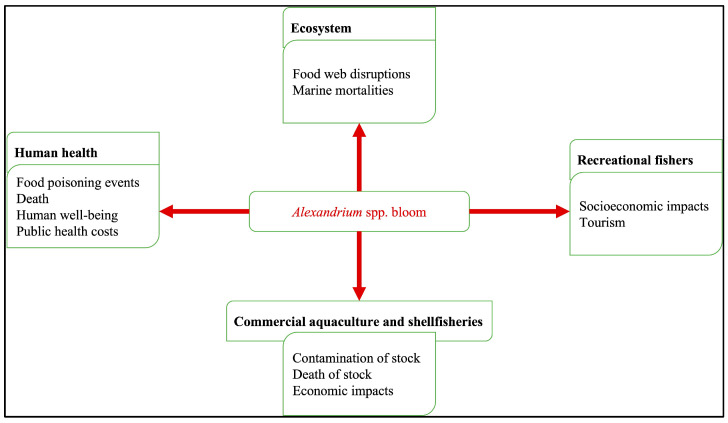
Impacts of *Alexandrium* spp. blooms.

Due to their complex life cycle, controlling blooms of *Alexandrium* is challenging [2]. This is mainly due to *Alexandrium* spp. forming resilient cysts that can remain dormant in sediments for prolonged periods and allow the microalgae to endure variations in environmental factors, such as fluctuations in temperature, salinity, or mechanical stress [16,17,18,19]. Measures to minimize the impact of *Alexandrium* blooms traditionally involve issuing advisories, imposing harvest, and sales restrictions, and implementing closures of aquaculture and shellfish farms [20]. Occasionally, the measures may extend to recalling exported shellfish after identifying toxin-producing taxa [2,21]. Additionally, impacts from *Alexandrium* bloom events (Figure 1) and the subsequent mitigation measures, such as temporary closures of shellfisheries, highlight the connection between environmental health and human activities [22,23] that lead to socioenvironmental impacts [24].

Recognizing the importance of identifying and quantifying *Alexandrium* during the pre-bloom period, many government and local authorities have initiated the development of early warning systems [21,25]. However, while monitoring can detect *Alexandrium* blooms and their toxins, actual removal of algal cells, or at least a reduction in the number of toxic cells, is necessary to minimize the impact of toxic blooms on fisheries, human health, and ecosystems [26]. The ideal approach to managing the impact of *Alexandrium* blooms should be an integrated one of early warning systems, strategies for the issuing of public health warnings, and the application of tools to remove or reduce bloom size. Such a management strategy requires cooperation across the environmental, public health, and socioeconomic sectors.

*Alexandrium* blooms can be problematic even at low densities. For instance, a bloom of *Alexandrium catenella* was recorded with a maximum density of 3 × 10^5^ cells L^−1^, reaching toxin levels up to 188 times higher than the maximum permissible limit of 0.8 mg STX di HCl eq/kg in blue mussels [27,28]. Such relatively low cell densities may render removal methods, such as clay application, ineffective. In contrast, within the Swan Canning Estuary, observed cell densities can reach as high as 1.5 × 10^7^ cells L^−1^, suggesting that treatment with clay could be a viable control strategy [29].

Removing microalgal cells from the water column using aluminosilicate clay-based products (bentonite, kaolinite, etc.) to create clay–algae flocs has quickly gained popularity as one of the preferred methods for treating harmful algal blooms in Korea [20,26], Australia [30], Japan [31], and China [32]. Improved flocculation efficiencies can be achieved with the co-application of natural clays with various floc-enhancing constituents, both inorganic (e.g., polyaluminum chloride (PAC) and ferric chloride) and organic (e.g., chitosan, betaine, and xanthan), as a potential alternative tool for HAB removal [33,34,35]. Laboratory-based studies of these “modified clays” have had varying degrees of success, contingent upon factors such as the type and dosage of clay utilized, the specific algal species involved, the modifier employed, the method of application, and the environmental conditions in the water column, such as pH and temperature, which may influence algal resuspension [36,37,38,39,40].

Despite the encouraging findings from previous laboratory studies on the use of PAC-modified clay to treat HABs, some studies have provided contradictory results, suggesting that further research is needed to determine the efficacy of flocculation when treating HABs. For example, a comparative study evaluating the treatment of the harmful algal bloom (HAB) species *Karlodinium veneficum* and *Karenia mikimotoi* using kaolin, zeolite, Korean loess, and six different bentonite clays found that bentonite clays consistently achieved the highest removal efficiency for both species [41]. These findings contradict a previously proposed theory suggesting that surface modifications would cause kaolinite clay to exhibit stronger Van der Waals attraction than bentonite clays [32]. This is attributed to the particle characteristics of kaolinite, its simpler structure, and weaker negative charges, which favor close-range interactions that lead to stronger Van der Waals attractions compared to bentonite clays [32]. It has also been reported that surface charge (zeta potential) varies depending on clay type and pH. At pH 7, kaolins exhibited the most negative potential among four clay types tested, and this potential decreased across all clay types when the pH was increased to 9 [42]. However, these results may reflect the differing characteristics (surface charge, size, shape, and motility) of the specific HAB species that were treated, which can also significantly influence the flocculation process [20,43,44,45]. These contrary findings indicate that it is crucial to analyze the underlying mechanisms that drive flocculation efficiency in order to propose, develop, and test modified clays that will have improved efficacy in the removal of HABs. The interaction between the microalgal cells and the nano-clay particles and their adhesion kinetics is governed by different factors such as surface charge, pH, and surface chemistry [46,47,48,49,50]. In particular, the surface properties of the clay play a particularly important role in the interactions and adhesion between the clay and algal cell surfaces [51], and this will be influenced by the surface and ion charge capacity of both the clay and algae, ultimately affecting the aggregation, adhesion, cohesion, and dispersion [52,53,54] of the flocs. The prediction of the adsorption and reaction behaviors of molecules on solid materials is important [55] and can be performed using DLVO theory or surface complexation models [56].

High HAB removal efficiencies have been achieved in laboratory studies through a combination of flocculation and settling when clay is applied as a suspension or slurry in distilled water [57,58]. Distilled water has been the preferred medium for these studies, as it appears to reduce the self-aggregation of clay particles prior to application [44,53,59]. In contrast, suspending clay in seawater tends to promote self-aggregation of clay particles, which can reduce removal efficiency [53]. However, for large-scale marine/estuarine applications, using seawater as a suspension medium can be more practical and cost-effective, despite its limitations. Thus, it is essential to determine the conditions under which removal efficiency is optimized when seawater is used as a suspension medium for producing the clay slurry.

Cell removal during flocculation can be affected by factors like cell density, treatment dose, the physical and chemical properties of the treatment, and environmental factors. As a result, the effectiveness of modified clay treatment in removing cells can vary greatly depending on the environment [32,60,61,62]. In the current study we used a local kaolinite clay with PAC. PAC was selected due to its cost-effectiveness and minimal environmental impacts [20,32,63]. The objective of this study was to assess the effectiveness of a local PAC–kaolinite clay in a seawater suspension for eliminating two toxigenic *Alexandrium minutum* strains found in estuarine environments in Western Australia. We analyzed the performance of the PAC clay in the laboratory using different clay doses, with and without PAC, at varying pHs (7 and 8) and with different initial cell densities. Additionally, a cost estimate was also conducted to provide insight into the economic feasibility of using kaolinite–PAC (KPAC) clay in comparison to kaolinite natural clay (KNAC).

## 2. Results

### 2.1. Removal Efficiencies

**Figure 2:**
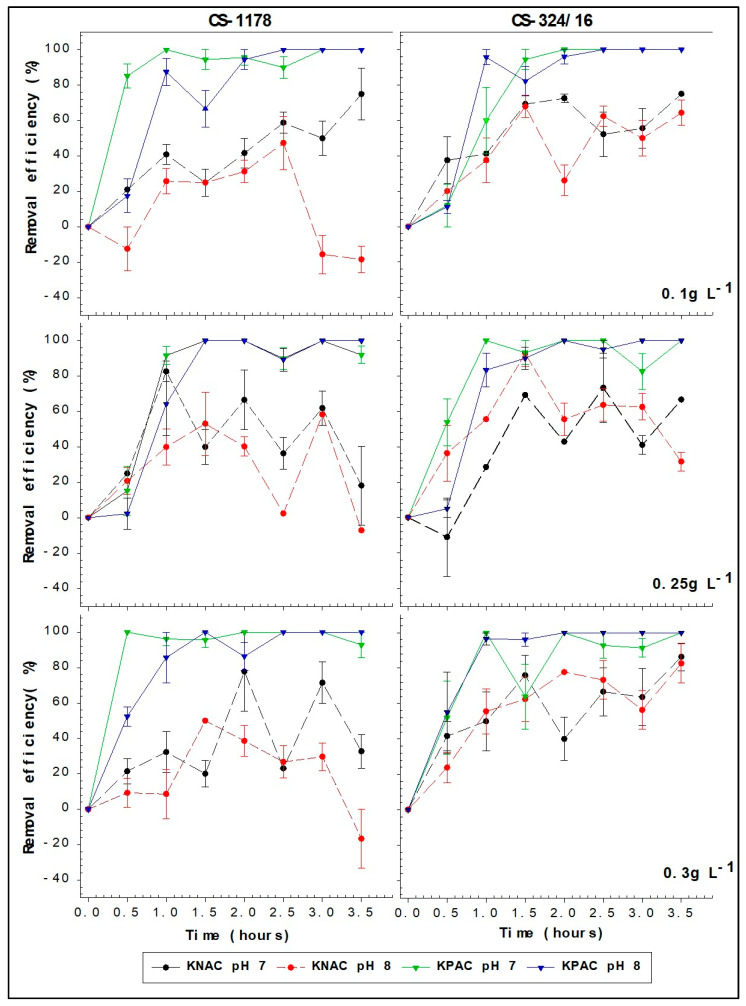
Removal efficiency of A. minutum strains CS-1178 and CS-324/16 with initial cell density of 1.0 × 10^7^ cells L^−1^. Cultures were treated with 3 different concentrations of KPAC (solid lines) and KNAC (dashed lines) at both pH 7 and 8.

**Figure 3:**
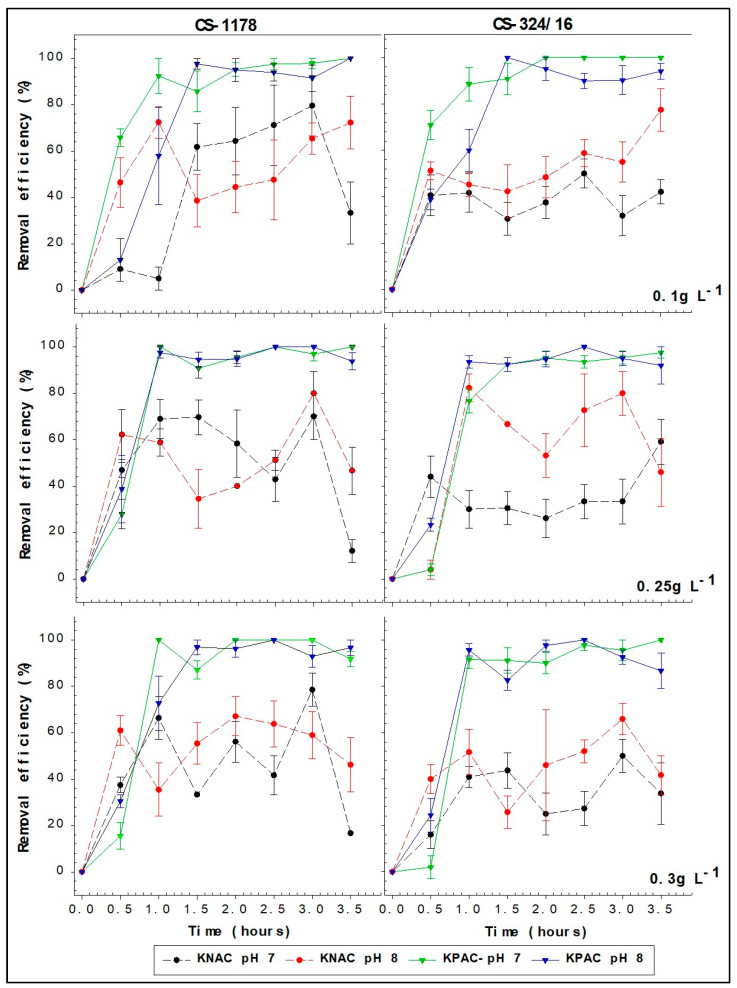
Removal efficiency of A. minutum strains CS-1178 and CS-324/16 with initial cell density of 2.0 × 10^7^ cells L^−1^. Cultures were treated with 3 different concentrations of KPAC (solid lines) and KNAC (dashed lines) at both pH 7 and 8.

Removal efficiencies in all kaolinite PAC clay (KPAC) treatments reached 100% within the first 2 h (Figure 2 and Figure 3). The maximum removal efficiencies in the kaolinite natural clay (KNAC) treatments were less than 85% and took longer to achieve (Figure 2 and Figure 3). Additionally, the removal efficiency for the natural kaolinite (KNAC) treatments decreased after the initial maximum, sometimes even falling below zero due to cell resuspension (Figure 2 and Figure 3). These differences in performance between the KPAC and KNAC treatments were significant, with the use of KPAC increasing the maximum removal efficiency (F_1,932_ = 185.0, *p* < 0.001 in experiment 1; F_1,932_ = 281.88, *p* < 0.001 in experiment 2) and decreasing the time to reach the maximum removal efficiency (F_7,932_ = 17.7, *p* < 0.001 in experiment 1; F_7,932_ = 34.58, *p* < 0.001 in experiment 2).

For the maximum removal efficiency, but not for the time to maximum removal efficiency, there was a significant interaction between the use of PAC and pH in experiment 1 (F_1,932_ = 6.55, *p* = 0.002) and between the use of PAC and clay concentration in experiment 2 (F_1,932_ = 1.83, *p* = 0.04). While the maximum removal efficiency was not affected by pH, strain, clay concentration, or initial cell density for those cultures exposed to KPAC in either experiment, for the KNAC treatments it was greater at pH 7 than at pH 8 in experiment 1 (1.0 × 10^7^ cells L^−1^), and it was greatest at a clay concentration of 0.25 g L^−1^, the mid-range concentration tested, in experiment 2 (2.0 × 10^7^ cells L^−1^).

The removal efficiencies calculated at 30 min intervals over 3.5 h of all KPAC treatments were adequately represented by the exponential rise equation r^2^ > 0.80 in all cases (Supplementary Figures S1 and S2). Estimated times to 95% removal efficiency when KPAC was tested varied between 0.79 and 2.37 h (Table 1). The curve fits for all KNAC treatments were much less reliable (r^2^ < 0.60).

**Figure 4:**
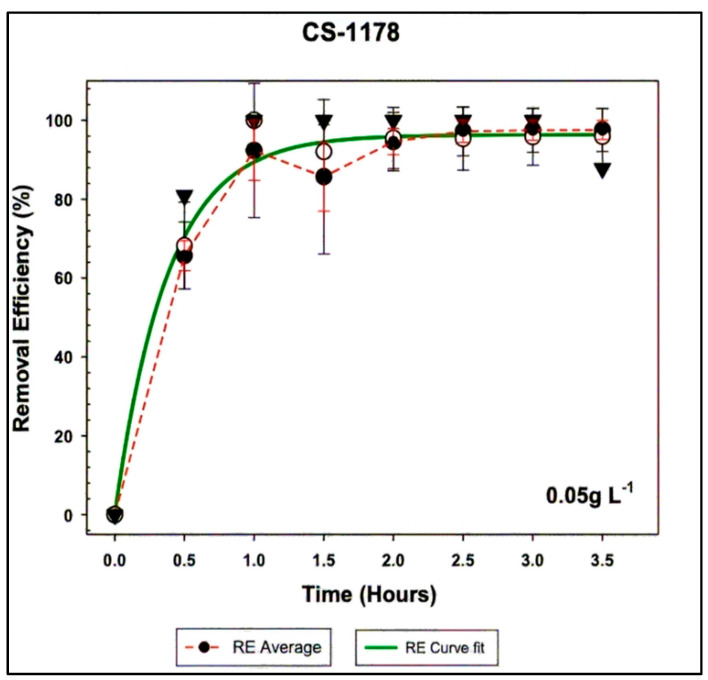
KPAC’s average removal efficiencies of *A. minutum* cells at a density of 2.0 × 10^7^ cells L^−1^ at pH 7, along with the corresponding fitted data curve.

The results achieved with the KPAC treatments led to the question of whether a lower concentration, i.e., <0.1 g L^−1^, could still yield high removal efficiencies in a reasonable time frame. To investigate this, a KPAC concentration of 0.05 g L^−1^ was used to treat *A. minutum* CS-1178 at a cell density of 2.0 × 10^7^ cells L^−1^. Removal efficiencies of up to 100% were observed within just 3 h of treatment. In addition, the curve fit analysis showed that a removal efficiency of 95% was achieved after 1.17 ± 0.16 h (Figure 4). However, the flocs formed during the experiment began to refloat after 2.5 h (Supplementary Figure S3).

### 2.2. Surface Charge

The zeta potential values for *A. minutum* CS-1178 and CS-324/16 were −7.03 mV and −7.23 mV, respectively. For KPAC slurry, the zeta potential at pH 7 was 10.53 mV; at pH 8, it was 5.70 mV; and at pH 9, it was 3.05 mV. For KNAC, the values were −17.57 mV at pH 7, −17.47 mV at pH 8, and −12.37 mV at pH 9.

## 3. Discussion

### 3.1. Removal Efficiency Mechanism

The addition of PAC significantly improved efficiency; PAC clay (KPAC) was effective against different strains at different starting algal concentrations, and efficiency was not affected by clay concentration or pH. Zeta analysis showed that PAC changed the surface charge of clay from negative to positive. The findings of this study are consistent with previous research (Table 2) that reported increased effectiveness when using PAC as a modifier for clay in the removal of harmful algal bloom species.

The efficiency of removing and treating HAB species using a clay is closely linked to the flocculation, deposition, advection, and resuspension characteristics [64]. The effectiveness of flocculation, in particular, is influenced by various factors, including chemical composition and surface properties [32,44]. These factors collectively contribute to the complex dynamics of movement and collision between algal and clay particles and the interparticle forces involved [20,43,44,45]; however, the surface properties of clay particles are the most critical factor determining flocculation efficiency [32].

One key surface property that is particularly important is the surface charge of clay/algal particles. Electrostatic interactions between particles can lead to either repulsive forces which occur when surfaces with the same charge come into contact [65], or attractive forces (Van der Waals attraction) when surfaces with different charges come into contact [32]. In this study, we hypothesized that the negative surface charge of both *A. minutum* and clay led to electrostatic repulsion when *A. minutum* cells were exposed to kaolinite natural clay (KNAC). Consequently, a larger quantity of clay may be required to achieve high removal efficiencies. For instance, previous studies have reported the use >380 t of unmodified natural kaolinite to treat a single square kilometer of a bloom caused by the red-tide-forming dinoflagellate *Cochlodinium polykrikoides* in Korea [20,32].

Many studies have suggested that limited initial contact between KNAC clay particles and algal cells hinders effective flocculation [43,44,66], leading to the formation of flocs with a lower settling velocity [67,68]. Flocs with a lower settling velocity result in two distinct effects. First, some cells can escape the floc structure and resume growth under suitable environmental conditions [40,67]. Second, there is reduced contact between clay and algal particles as a result of hydrodynamic retardation. This occurs when algal cells with a smaller mass find it difficult to approach larger particles (clay–algae flocs) due to the hydrodynamic forces generated by the water displaced by the much larger floc particle areas as they descend through the water column [40,44,59,64]. This may explain the resuspension of flocs in the water column observed when the KNAC treatment was applied to *A. minutum* in our study. Resuspension of flocs results in a negative removal efficiency being calculated. A similar phenomenon has been observed using a different type of kaolinite clay (SWE2), suggesting that the clay “stabilized” the cell culture and prevented natural sinking of *Prymnesium parvum* cells, thereby limiting effective removal [40]. In the present study, it is likely that *A. minutum* cells treated with KNAC remained viable and continued photosynthesizing, producing oxygen that caused the flocs to float (resuspend). This theory has also been reported in [40].

Resuspension of *A. minutum* was not observed following the application of kaolinite–PAC clay (KPAC). This suggests that the addition of PAC modified the surface charge of kaolinite from negative to positive, resulting in the entrapment of *A. minutum* cells within the flocs and consequently reducing their viability. This suggests that PAC increased the clay’s affinity for algal cells through Van der Waals attraction, as confirmed by zeta potential analysis, thus enhancing flocculation by producing larger/denser flocs [32,33,44,69]. The PAC clay then acted as ballast in these large flocs, resulting in comparatively rapid sinking and sedimentation of the HAB cells [66] and minimizing opportunities for resuspension.

There have been suggestions that the pH of a culture is critical to its removal efficiency, with the cellular activity of *Alexandrium* cells significantly influencing lower removal efficiencies. However, it has been reported that the growth of *Alexandrium catenella* cells is not universally affected by lower pH but decreases when combined with elevated temperatures (20 °C), suggesting an influence on cellular activity due to the temperature being maintained at 16 °C during the experiment [70]. Thus, the possibility of pH affecting the natural sinking of cells can be ruled out. The findings of this study are consistent with previous research (Table 2), which reported effectiveness when using PAC as a modifier for clay in the removal of harmful algal bloom species.

**Table 2:** Variations in removal efficiencies in PAC-modified clay treatment studies.

Clay Type	Clay (gL^−1^)	Cell Density (Cells L^−1^)	Removal Efficiency	Tested Species	Ref.
Kaolinite clay (unmodified)	1	-	80%	*Noctiluca scintillans* *Prorocentrum minimum*	[70]
Yellow loess modified with biosurfactant sophorolipid ratio (1:5)	10	1.3 to 2.1 × 10^6^	95%—after 30 min	*Cochlodinium polykrikoides*	[71]
Kaolin with PAC ratio (1:5)	0.5	8 to 9 × 10^9^ & 2.2 to 2.5 × 10^9^	80–82%	*Aureococcus anophagefferens* *Phaeocystis globosa*	[48]
MC II (kaolin with PAC) ratio (5:1)	0.2	1.0 × 10^6^	57% after 8 h 95% after 48 h	*Karenia brevis*	[53]
MC II (kaolin with PAC) ratio (5:1)	0.5	1.0 × 10^6^	95% after 24 h	*Karenia brevis*	[55]
Kaolin with PAC ratio (5:1)	0.3	3.0 × 10^6^	83.67%	*Karenia mikimotoi*	[72]
Dry bentonite with PAC ratio (1:2)	0.5	1.9 × 10^8^	50%	*Prymnesium parvum*	[40]
Wet bentonite with PAC ratio (1:2)	0.1	1.0 × 10^3^	100%	*Prymnesium parvum*	[40]

While improvements in removal efficiencies are acknowledged when using PAC-modified clays, the efficacy may vary depending on the clay type and structure, algae species, and environmental factors. Historically, researchers considered clay mineral group montmorillonites to have the best removal efficiencies due to their three-layered (-Si-Al-Si-) structure, in contrast to the two-layer (-Al-Si-) structure of kaolinites [32,44]. These structural differences between montmorillonite and kaolinite affect their interaction with HAB cells. Montmorillonite has a higher negative surface charge due to greater isomorphic substitution, but its expandable layers promote aggregation, reducing dispersion [32]. In contrast, kaolinite’s stable, non-expandable structure minimizes aggregation, enhancing its effectiveness in HAB mitigation despite its moderate surface charge [32]. Interestingly, researchers reported differences in clay structures within the same type of clay from different geographical areas. For example, in Japan, kaolinite clay was found to be an ineffective flocculant in seawater and was observed to have similar layers to montmorillonite clay sourced from China [31,71]. These differences prompted further investigation into the effectiveness of these clays in removing algal cells [32,68,71,72]. These studies suggest that the type of clay and layers alone do not determine HAB removal efficacy, but that the interactions of the clay in freshwater and seawater, as well as the specific types of algae involved, also need to be considered. Therefore, while the efficacy of unmodified clays cannot be accurately predicted, PAC-modified clays show more consistent results across all regions, clay types, and HAB species.

The base medium of the clay slurry applied to bloom-infested waters has been shown to significantly impact removal efficiencies. Studies have reported removal efficiencies when PAC was used in a seawater suspension compared to fresh water [44,58,59,73]. For example, achieving 80% removal of *Aureococcus anophagefferens* required 0.6 g L^−1^ PAC-modified clay prepared in seawater, but only 0.2 g L^−1^ was required when prepared with fresh water to achieve the same removal efficiency [58]. It has been suggested that the low removal efficiencies observed when using seawater as the suspension medium may be due to its relatively high concentrations of polyvalent ions, particularly negatively charged sulfates, which can reduce the surface charge of the modified clay [58]. Low removal efficiencies when using seawater as a clay suspension medium have also been attributed to the higher pH (>8) of seawater slurries, which can decrease the surface charge of the modified clay, contributing further to the low removal efficiencies of the modified clay [44,58,59,71,73]. However, the current study showed high removal efficiencies using a lower KPAC concentration of 0.1 g L^−1^ in a seawater suspension. This is a critical cost-saving measure when applying clay in marine waters, as site water can be used to prepare the clay suspension, obviating the need to purchase and manage large quantities of fresh water.

Stirring can also impact removal efficiency. For instance, it has been hypothesized that interparticle interactions are enhanced under mixing conditions and therefore occur more frequently in turbulent aquatic environments [59]. However, the current study did not incorporate mixing, as it was designed to simulate a large-scale clay application in natural water bodies. The underlying assumption was that the water would remain still during application, with no wind or other external disturbances.

The impact of cell density on removal efficiency can also be substantial. Previous studies have reported high removal efficiencies at low cell densities (Table 2). For instance, a maximum removal efficiency of 100% was achieved using PAC-modified wet bentonite to remove *Prymnesium parvum* cells at the lowest tested density of 1.0 × 10^6^ cells L^−1^ [40]. Additionally, the researchers highlighted that besides cell concentrations, clay concentrations also play a crucial role in impacting removal efficiency [40]. In contrast, the results of this study revealed no significant difference in removal efficiencies between the various concentrations of KPAC (0.1, 0.25, and 0.3 g L^−1^) used across the two cell densities tested (1.0 and 2.0 × 10^7^ cells L^−1^). This led to trials with lower concentrations of KPAC (0.05 g L^−1^). High removal efficiencies of over 95% were achieved; however, the settled flocs formed begun to float after 2.5 h. We hypothesize that, at this application rate, the floating could be due to the low density of the clay–algae floc being insufficient to counter cellular buoyancy from lipids and production of oxygen (microbubbles in the clay–cell matrix). In addition, in field application situations, the flocs can be disrupted with wave turbulence, lowering the removal efficiency. These results suggests that each clay–algae interaction is unique and should be addressed individually.

### 3.2. Ecological and Environmental Impact

Potential environmental impacts, particularly on benthic and aquatic environments, also need to be considered when using modified clays, such as KPAC, to treat algal blooms. While the clay loadings are significantly reduced in comparison to using unmodified clay, there are still impacts associated with clay loadings in the benthic environment. Clay treatment can have significant ecological impacts, especially on species that produce cysts, such as *A. minutum*. One concern is the potential formation of cyst beds, which could create optimal conditions for cyst production [74,75]. However, treatment with modified clay has been shown to not only reduce the germination rates of these cysts but also to inhibit their formation [76,77]. These findings indicate that while initial concerns exist, modified clay may ultimately reduce cyst viability and lower the ecological risks associated with cyst formation. In addition, there are concerns about aquatic animals when using PAC in modified clay treatments. Recent studies have shown that PAC levels below 0.5 g L^−1^ have little to no impact on the mortality, survival, or reflexes of several aquatic animals [32,63,78,79,80]. Therefore, it is reasonable to consider concentrations of PAC clay of 0.1 g L^−1^ as desirable for treating blooms due to negligible impacts on benthic and aquatic environments. In the current study, high removal efficiencies of 100% were achieved at KPAC concentrations of 0.05 g L^−1^ and 0.1 g L^−1^, classifying it as environmentally friendly.

### 3.3. Cost Estimate

To be useful, treatments for HABs need to be cost-effective. We have undertaken a preliminary comparative analysis of the cost of removing *Alexandrium* using natural clay and PAC clay. A PAC clay (KPAC) concentration of 14.08 kg ha^−1^ was based on the laboratory results from this study, while a natural clay (yellow loess) concentration of 384 t km^−2^ reflects a successful large-scale application rate for natural clay in Korea [20,32,71]. Costs were compared against potential impacts for various historical bloom events (Supplementary Table S1). Cost estimates for large-scale treatments were based on three assumptions: laboratory results would scale linearly; the surface area of the measuring cylinders was proportional to the bloom area; and treatment concentration would increase linearly with bloom density above 2.0 × 10^7^ cells L^−1^, using this density as a baseline.

The cost estimates are summarized in Table 3. All costs are presented in USD and, if necessary, were converted from local currency using https://www.rba.gov.au/ (accessed on 25 January 2025). Material costs referenced the cost of kaolinite and PAC. The operational cost of using KPAC was 11.1% that of utilizing natural clay on a per-hectare basis (Table 3). When compared to the application of KNAC, the individual category costs of treating a bloom with KPAC were dramatically reduced: labor (21.9% of KNAC cost), slurry materials (clay + PAC, 5.2%), storage (7.3%), transport (3.9%), and boat use (59.6%). These large reductions in operating costs are due to the much lower amounts of clay slurry needing to be dispersed, thus allowing the use of a smaller boat, reduced transport and storage costs, and less time on the water. Transport costs are the largest single operational expense. These transportation costs can become economically unfeasible if kaolinite and PAC are not sourced locally or are not readily available [67]. The estimated cost was based on the maximum density of *Alexandrium* cells recorded during historical bloom events, assuming that the bloom covered the entire area.

Purchase of a dispenser is a fixed capital cost and is required for both scenarios, but it can be used across multiple blooms and years. Even assuming that the dispenser was only used once, the cost per hectare for treating an *Alexandrium* outbreak with KPAC is still 68% of the same treatment with KNAC. Our preliminary cost estimates indicated that applying KPAC to a large-scale *A. minutum* bloom would be more cost-effective than using unmodified clay. Additionally, the use of natural clay requires significant amounts and raises concerns about potential environmental impacts.

The cost-effectiveness of using PAC clay for treatment should account for the broader economic impacts experienced during various *Alexandrium* spp. bloom events. These impacts depend on the specific usage of the affected waterway; for example, recreational fishing, commercial shellfisheries, aquaculture, ecosystem services, and cultural significance can affect the arguments about how to treat a bloom. The use of clay for treatment is well established in the aquaculture industry [20], primarily due to its cost-effectiveness in removing toxic algal blooms. That said, there are advantages to treating these blooms in non-commercial locations, provided that the costs are not prohibitive. These advantages extend beyond commercial fisheries and recognize the environmental value of the affected area.

*Alexandrium* bloom events typically occur during the peak spring and summer seasons, characterized by high tourism, recreational, and commercial activities, which can result in high demand for aquaculture products [82]. For instance, treatment options can easily be considered in areas such as Syracuse, Italy [83]; Crique-de-l’Angle, France [75]; and approximately 20% of the Thau lagoon, France [74,75] (Supplementary Table S1), which are areas dedicated to commercial shellfish farming and aquaculture. In these cases, their economic impacts are easily quantifiable risks that can be measured against the cost of mitigation since data on positive economic impacts are readily available [84]. On the contrary, in most recreationally intensive areas such as Hunts Bay, Jamaica [85,86], and the North Lake of Tunis, Tunisia [87], there may be a lack of positive economic impact data, making the decision-making process difficult.

Decision-making can also involve other factors when there is a lack of positive economic data. One factor is considering the deficiencies in existing warning systems that undermine their intended purpose and potentially lead to unintended consequences, for instance, in preventing large-scale health issues that could cause community social welfare losses. These health issues arise from relying on recreational fishers’ willingness to comply with rules regarding closed areas [88]. Second, there may be impacts on the local tourism economy due to reduced spending in local businesses due to a decline in or halt in fishing [88,89]. Third, closures risk cutting off access to resources depended upon by some families. Finally, consideration should also be given to the cultural ecosystem services (i.e., aesthetic, and inspirational value and spiritual and indigenous significance). Temporary closures can impact human well-being, disrupting opportunities for therapeutic and inspirational experiences in the natural environment [23]. For example, previous studies have reported that pollution can hinder recreational opportunities and contribute to the loss of a community’s traditional way of life, sense of place, and collective identity [23,89].

Finally, decision-making should also consider different *Alexandrium* species that exhibit varying toxicity levels. This can result in different environmental costs, such as fish kills and shellfish contamination. These variations can have specific economic impacts and public health costs [12]. Therefore, it is essential to understand the specific impacts of each species in order to assess the cost-effectiveness of treatment. Essentially, decisions on whether to treat or not treat algae should compare removal costs to all potential impacts and impending environmental costs. Mitigation measures should minimize the overall costs, encompassing both economic losses and expenses associated with mitigation efforts. Striking a balance is essential, as society may need to accept certain losses to maintain lower overall costs [90].

## 4. Conclusions and Future Directions

This study found that kaolinite–PAC clay (KPAC) achieved high removal efficiencies (>90%) when applied to toxic *Alexandrium minutum* suspensions of varying densities and pH levels. Importantly, these high removal efficiencies were accomplished using saline site water as the clay suspension medium. Zeta potential measurements showed that PAC changed the surface charge of kaolinite from negative to positive, working to enhance both electrostatic interactions and flocculation efficiency.

Clay-based flocculation of HABs is considered one of the most favored control options in open waters and has been applied extensively to protect economic interests such as commercial fisheries. Both the cost and concerns regarding negative impacts on benthic environmental values have meant little to no uptake in other scenarios. KPAC’s high removal efficiency with low-concentration slurry (0.1 g L^−1^) leads to significant positive advances regarding both cost and reduced potential environmental impacts. While this opens the door for potential wider application of PAC clays to control HABs and greater protection of cultural and recreational fishing activities, as well as environmental values, further research is needed in a large-scale mesocosm trial to accurately represent field conditions.

A mixed management approach is often most successful for the management of harmful algal blooms, such as those of *Alexandrium minutum*. This entails integrating monitoring, preventative management, and public education ahead of response-type mitigation actions such as the application of modified clays. Additionally, it is crucial to continuously evaluate any environmental impacts of any applied mitigation action. Finally removing blooms at their early stages proves to be a more cost-effective approach due to lower densities of *Alexandrium* cells, smaller area coverage, and lower treatment concentrations. However, comprehensive economic assessments, inclusive of wider socioeconomic impact across both the short and medium term, are essential for determining optimal management strategies.

## 5. Materials and Methods

### 5.1. Removal Efficiency

#### 5.1.1. Clay Preparation

Commercial kaolinite clay (WA Kaolin, Perth, Western Australia, Australia) was used as the base material for preparing two stock solutions of clay slurries: kaolinite natural clay (KNAC) and kaolinite clay with the addition of polyaluminum chloride or PAC (KPAC). To prepare KNAC, 2.5 g of kaolinite was mixed with 100 mL of filtered seawater using a vortex mixer, giving a final concentration of 25 g L^−1^. The KPAC slurry contained 1:1 *w*/*w* kaolinite clay and PAC (Pacific Water Technology, Brisbane, Australia): 2.5 g of kaolinite clay and 25 mL of 10% aqueous PAC (equivalent to 2.5 g PAC) mixed with 75 mL of filtered seawater.

#### 5.1.2. *Alexandrium minutum* Stock Culture Preparation and Maintenance

Two pure strains of *Alexandrium minutum* were obtained from the culture collection of the Australian National Algae Supply Service, Commonwealth Scientific and Industrial Research Organization: CS-1178 (isolated from Bunbury, Western Australia, Australia) and CS-324/16 (isolated from Port River, South Australia, Australia). Both strains were cultured using GSE/2 modified media [91], with a salinity of 32 ± 1 ppt. Cultures were maintained at a temperature of 16 ± 1 °C using a 12:12 h dark/light cycle and a photon flux of 60–70 μmol photon m^−2^ s ^−1^. Cultures were gently mixed by hand and monitored every second day to assess growth and contamination using a Neubauer hemacytometer and a Leitz Laborlux 12 microscope.

#### 5.1.3. Experimental Design

Two separate experiments were conducted to measure the efficacy of clay treatment in removing *A. minutum* cells from solution: one at 1.0 × 10^7^ cells L^−1^ (experiment 1) and one at 2.0 × 10^7^ cells L^−1^ (experiment 2). Final cell density was verified by cell counts with a Neubauer hemacytometer [92]. The pH 8 treatment was selected, as it generally represents the marine part of the Swan Canning Estuary during the summer season when *Alexandrium* blooms. However, *Alexandrium* has been observed blooming up to approximately 45 km upstream, where the pH can be as low as ~7.15 during the bloom season [93]. In addition, the pH is generally more variable (and lower) in waters near the benthos due to biogeochemical processes [94]. Therefore, a slightly lower pH of 7 was chosen to account for conditions that might be experienced by the clay flocs when approaching the bottom.

For each experiment, a factorial design was implemented, considering four factors: strain (CS-1178; CS-324/16), pH (7 and 8), clay concentration (0.1, 0.25 and 0.3 g L^−1^), and use of PAC. Cultures were pH-adjusted (from a starting pH of 7 ± 1) using NaOH and HCl, gently mixed by hand to ensure the homogeneous distribution of algal cells, and distributed into five replicates, with 100 mL graduated measuring cylinders assigned to a particular treatment combination. To account for natural sinking of *A. minutum* cells, a control group (comprising 5 replicates) was included in each treatment group. The control comprised an untreated culture with seawater (32 ± 1 ppt) added in a volume comparable to that of the clay treatment (to account for potential dilution effects). Clay suspensions were applied to the surface of the *Alexandrium* within the graduated measuring cylinders utilizing a 100–1000 μL and 2000 μL variable volume micropipette (Supplementary Table S2). The environmental conditions throughout each experiment were maintained at 16 °C ± 2 °C, and the light intensity was maintained between 60 and 70 μmol photon m^−2^ s^−1^.

The total duration of each experiment was 3.5 h, with sampling of 1 mL aliquots (removed from the 50 mL mark of the cylinder) at half-hour intervals. The 1 mL samples from each replicate at the different time points was immobilized with 200 μL of Lugol’s iodine, and cells were counted using a Neubauer hemacytometer and a Leitz Laborlux 12 microscope [92]. Removal efficiencies were then calculated following (1) [44].

(1) $$Removal\;Efficiency\;\%=1-\frac{Final\;cell\;density\;}{Mean\;final\;density\;of\;control\;}\times 100$$

#### 5.1.4. Data Analysis

Removal efficiencies were plotted over time for each treatment combination for the KNAC and KPAC treatments. Maximum removal efficiency and time to maximum removal efficiency were calculated for each replicate and analyzed separately for each experiment (i.e., for each starting concentration of algal cells) using general linear models, with fixed effects of clay concentration, pH, presence/absence of PAC, and all interactions among these predictors, with algal strain treated as a random effect. Maximum removal efficiency was arcsine-transformed, and normality assumptions were confirmed with residual plots following all analyses. Statistical analyses were performed using R version 4.3.2 (R Core Team, Vienna, Austria, 2024) on macOS.

Time to achieve 95% removal efficiency was used as a summary measure of effectiveness for all treatment combinations. This was estimated from curves fitted to the raw data using the equation Y = a (1 − b^x^), where a is the maximum removal efficiency, b is the rate of change in the exponential function, and x is the time (h). In order to ensure that the fitted curve adequately reflected the settling process, the data coefficient of determination (R^2^) was required to be 0.8. The graphing of this were performed using SigmaPlot version 15.0 (Systat Software Inc., San Jose, CA, USA, 2023).

### 5.2. Surface Charge Analysis

The zeta potential of both KNAC and KPAC was measured in 3 replicates using a Malvern Panalytical Zetasizer lab. The slurries were thoroughly mixed, and the samples were prepared by taking ~3 g of the clay and dispersing them in 30 mL deionized water (samples needed to be highly diluted for the measurement). NaCl was added to achieve 10 mM background electrolyte, and the pH was adjusted to 7–9 with NaOH and/or HCl, which corresponds to the pH range when *Alexandrium* blooms in the Swan Canning River, Perth Western Australia.

## Figures and Tables

**Table 1:** Outcome of *A. minutum* CS-1178 and CS-324/16 removal efficiency using PAC-treated clay (KPAC), showing the calculated times for 95% removal efficiencies based on the fitted exponential curves. The top three fastest times are represented by superscripts a, b, and c; the bottom three slowest times are represented by superscripts x, y, and z.

*A. minutum* Species	Cell Density	pH	0.1 g L^−1^	0.25 g L^−1^	0.3 g L^−1^
			Time (Hours)		
CS-1178	1.0 × 10^7^ cells L^−1^	7	0.62 ± 0.04 ^b^	1.50 ± 0.22	0.05 ± 0.04 ^a^
		8	2.58 ± 0.80 ^y^	2.42 ± 0.78 ^x^	1.53 ± 0.22
	2.0 × 10^7^ cells L^−1^	7	1.31 ± 0.16	2.04 ± 0.45	2.36 ± 0.55
		8	2.15 ± 0.64	2.10 ± 0.36	2.04 ± 0.37
CS-324/16	1.0 × 10^7^ cells L^−1^	7	2.03 ± 0.80	1.23 ± 0.17	0.88 ± 0.13
		8	0.71 ± 0.06 ^c^	2.27 ± 0.60	0.71 ± 0.06 ^c^
	2.0 × 10^7^ cells L^−1^	7	1.65 ± 0.29	1.57 ± 0.24	2.10 ± 0.56
		8	1.37 ± 0.15	2.70 ± 2.89 ^z^	2.21 ± 0.15

**Table 3:** Cost estimate comparison of kaolinite–PAC clay (KPAC) and natural clay for 90% removal of *Alexandrium minutum* in a one-hectare bloom scenario.

	Kaolinite PAC Clay (KPAC—140.8 kg ha^−1^)	Kaolinite Natural Clay (Yellow Loess—3840 kg ha^−1^)
	USD Per ha ^1^	
Labor ^2^	46.20	211.23
Clay ^3^	11.26	614.00
PAC ^4^	20.49	-
Storage ^5^	4.44	60.48
Transport ^5^	48.29	1232.85
Boat per day ^6^	185.85	311.85
Operational costs	316.53	2430.41
Dispenser ^7^	4300	4300
Operational + fixed costs	4616.53	6730.41

^1.^ These cost estimates are solely based on preliminary figures and do not account for inflation or unforeseen logistical changes. It is acknowledged that costs are representative of the Australian market and may vary based on the actual market prices in different countries and actual implementation time. As such, the figures should be interpreted as indicative rather than comprehensive. ^2.^ Technician labor cost (USD 23.47 per h) was multiplied by the required staff members; the costs reflect wages in an Australian context. Staff levels (2.13) were based on estimation of data from [20] for the absolute minimum required to spread a ton of clay. The labor cost was based on technician salary (https://au.talent.com/ (accessed on 25 January 2025)) and represents the costs for 2025. ^3.^ The cost of kaolinite clay used was USD 0.16 per kg, as reported by the Statistical Research Department in the USA (https://www.statista.com/statistics/ (accessed on 25 January 2025). ^4.^ The cost of PAC was USD 0.29 per kg, as reported by Yixing Bluwat Chemicals Company (https://www.waterpurifyingchemicals.com/ (accessed on 25 January 2025)). ^5.^ Transport costs were sourced from a local kaolin manufacturer, WA kaolin (https://wakaolin.com.au/ (accessed 25 January 2025) at USD 343, based on their 2025 prices, and storage costs of USD 31.50 per t were calculated using a parametric rule, informed by data presented in [20]. ^6.^ Per-day boat rental rates in Perth (https://www.boatingwest.com.au/ (accessed on 25 January 2025)): small boat: 4.27 × 1.90 m (L × W) at USD 295 per day; larger boat: 6 × 2.48 m (L × W) at USD 495 per day. ^7.^ The cost of dispensing considered the purchase of a dispenser [81].

## Data Availability

The original contributions presented in this study are included in the article and Supplementary Material. Further inquiries can be directed to the corresponding author.

## Supplementary Materials

The following supporting information can be downloaded at: https://www.mdpi.com/article/10.3390/toxins17080395/s1, Figure S1: Curve fitting of removal efficiencies of *A. minutum* strains CS-1178 and CS-324/16 with initial cell density 1.0 × 10^7^ cells L^−1^. Cultures were treated with 3 different concentrations of KPAC at both pH 7 (solid line) and 8 (dashed line). Figure S2: Curve fitting of removal efficiencies of *A. minutum* strains CS-1178 and CS-324/16 with initial cell density 2.0 × 10^7^ cells L^−1^. Cultures were treated with 3 different concentrations of KPAC at both pH 7 (solid line) and 8 (dashed line). Figure S3: Image of treatment with 0.05 g L^−1^ of KPAC showing floating flocs at hour 2 and 3.5 of the experiment in comparison to control. Table S1: Comparison of material cost estimates for 0.1 g L^−1^ KPAC (PAC + Clay) and 384t km^−2^ yellow clay for treatment of *Alexandrium* bloom across historical scenarios. Table S2: Final clay concentrations calculations using surface area.

## Abbreviations

The following abbreviations are used in this manuscript:

MC	Modified clay
KPAC	Kaolinite–polyaluminum chloride clay
KNAC	Kaolinite natural clay
RE	Removal efficiency
WA	Western Australia
PAC	Polyaluminum chloride
HAB	Harmful algal bloom
PST	Paralytic shellfish toxin
NaCl	Sodium chloride
NaOH	Sodium hydroxide
HCL	Hydrochloric acid
