# Supplementary material for: Effectiveness of Kaolinite with and Without Polyaluminum Chloride (PAC) in Removing Toxic Alexandrium minutum
Source: Toxins (Basel). 2025 Aug 6;17(8):395. doi: 10.3390/toxins17080395 (PMC12390390; doi:10.3390/toxins17080395)
Supplement: Supplementary file 1 [file toxins-17-00395-s001.zip › toxins-3733094-supplementary.pdf]

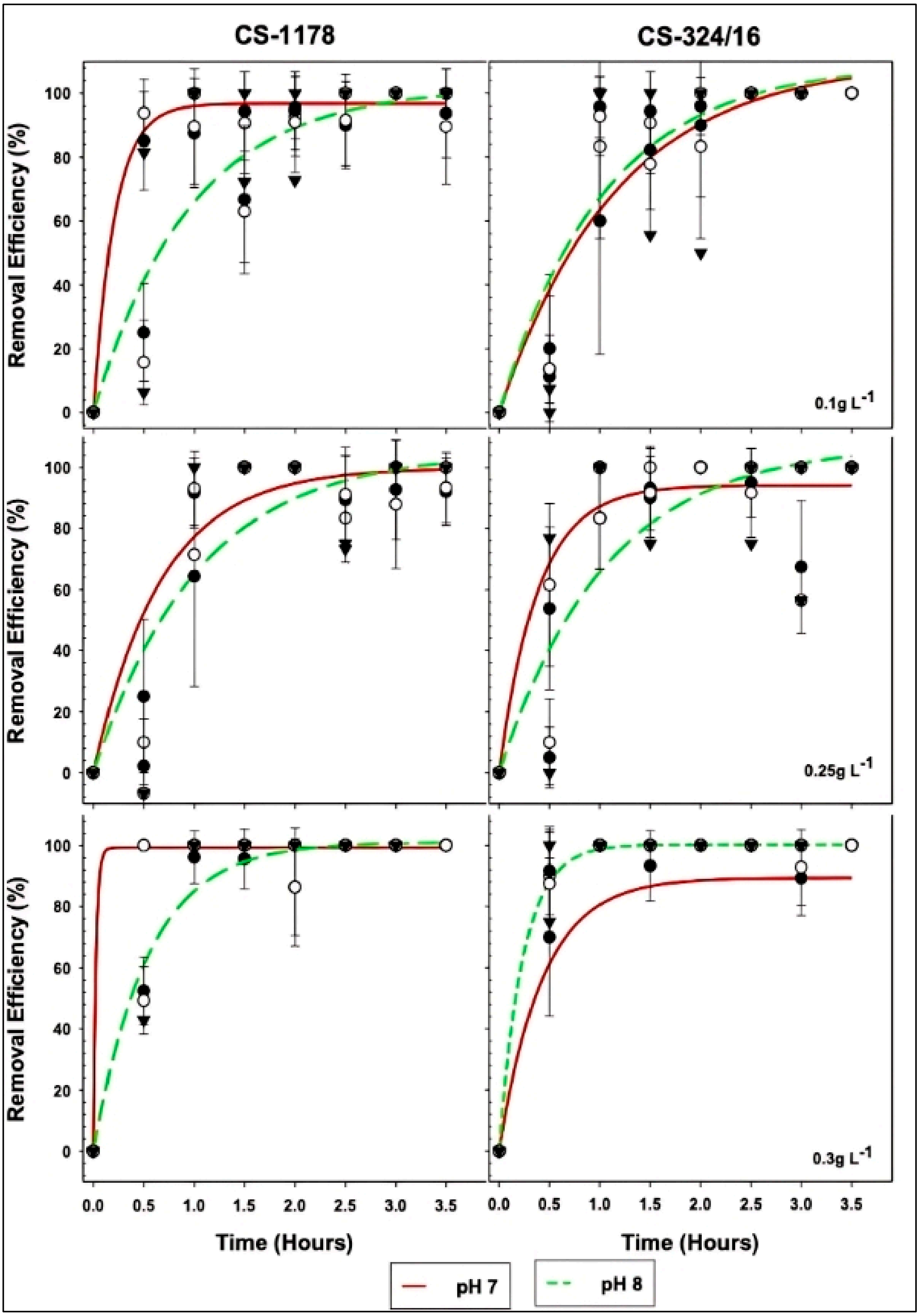

Supplementary Figure S1. Curve fitting of removal efficiencies of *A.minutum* strains CS-1178 and CS-324/16 with initial cell density 1.0 x 10^7 cells L^{-1}. Cultures were treated with 3 different concentrations of KPAC at both pH 7 (solid line) and 8 (dashed line).

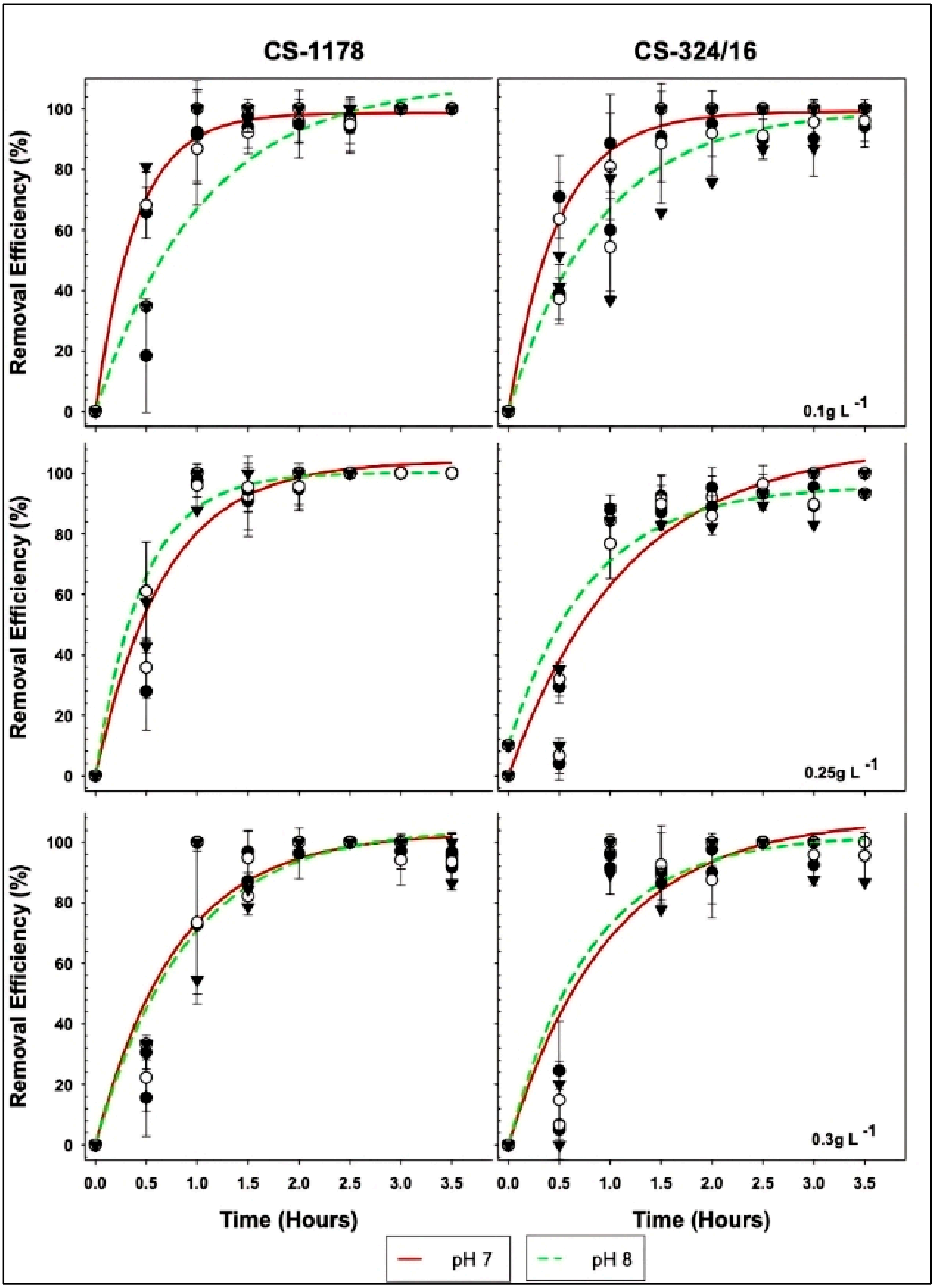

Supplementary Figure S2. Curve fitting of removal efficiencies of *A.minutum* strains CS-1178 and CS-324/16 with initial cell density 2.0 x 10^7 cells L^{-1}. Cultures were treated with 3 different concentrations of KPAC at both pH 7 (solid line) and 8 (dashed line).

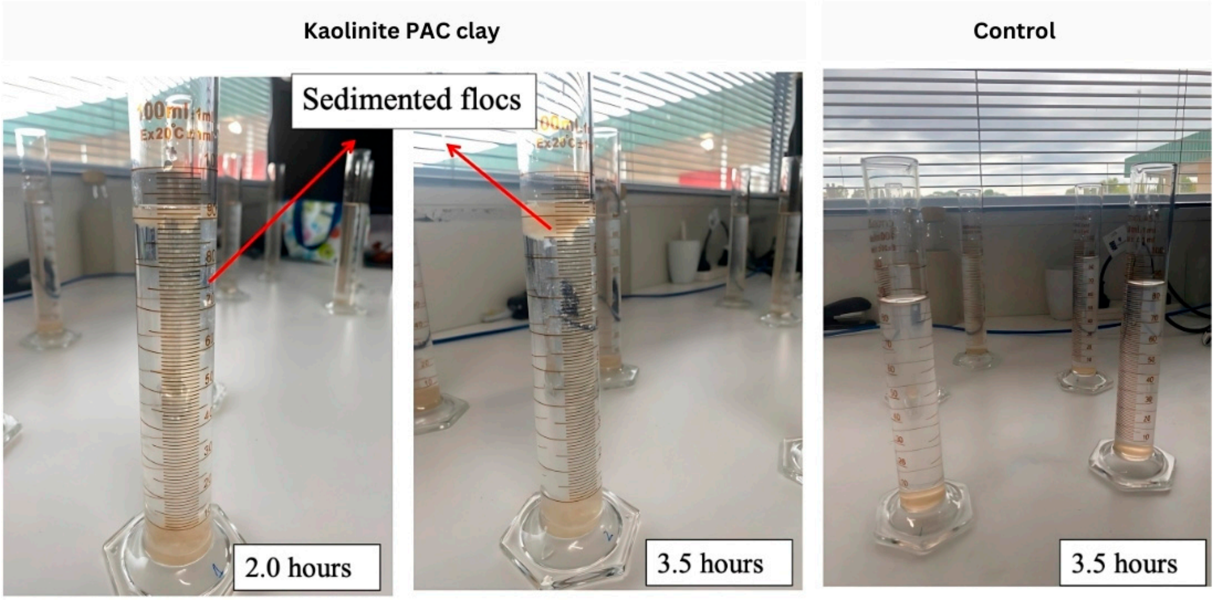

Supplementary Figure S3. Before (Time-0) and after (Time 3.5) images of KPAC, KNAC and Control treatments of *Alexandrium minutum* (CS324/16).

Supplementary Table S1. Final concentrations calculations using surface area

**Kaolinite Natural Clay (KNAC)**	**Kaolinite PAC (KPAC)**
Stock solution-25g/L	Stock solution-25g/L
Kaolinite(2.5g) + seawater (100mls	Kaolinite(2.5g) + PAC (25mls) + seawater (75mls)
**Concentration calculations**	
**0.1 gL-1**	
$C_1V_1 = C_2V_2$ $25gL^{-1}, x = 0.1gL^{-1}, 100ml$ $25gL^{-1}\,(x)25gL^{-1} = 0.1gL^{-1}, 100ml$ $\frac{25gL^{-1}\,(x)}{25gL^{-1}} = \frac{0.1gL^{-1}, 100ml}{25gL^{-1}}$ $x = \frac{0.1 \times 100ml}{25g}$ $x = 0.4ml$	
**0.25 gL-1**	
$C_1V_1 = C_2V_2$ $25gL^{-1}, x = 0.25gL^{-1}, 100ml$ $25gL^{-1}\,(x)25gL^{-1} = 0.25gL^{-1}, 100ml$ $\frac{25gL^{-1}\,(x)}{25gL^{-1}} = \frac{0.25gL^{-1}, 100ml}{25gL^{-1}}$ $x = \frac{0.25 \times 100ml}{25g}$ $x = 0.6ml$	
**0.3 gL-1**	
$C_1V_1 = C_2V_2$ $25gL^{-1}, x = 0.3gL^{-1}, 100ml$ $25gL^{-1}\,(x)25gL^{-1} = 0.3gL^{-1}, 100ml$ $\frac{25gL^{-1}\,(x)}{25gL^{-1}} = \frac{0.3gL^{-1}, 100ml}{25gL^{-1}}$	

$$x = \frac{0.3\ \times 100ml}{25g}$$

$$x = 1ml$$

0.05 gL^{-1}

$$C_1V_1 = C_2V_2$$

$$25gL^{-1}\ , x = 0.05gL^{-1}\ , 100ml$$

$$25gL^{-1}\ (\ x)25gL^{-1}\ = 0.05gL^{-1}\ , 100ml$$

$$\frac{25gL^{-1}\ (\ x)}{25gL^{-1}} = \frac{0.05gL^{-1}\ , 100ml}{25gL^{-1}}$$

$$x = \frac{0.05\ \times 100ml}{25g}$$

$$x = 0.2ml$$

Supplementary Table S2. comparison of material cost estimates for 0.1g L $^{-1}$ KPAC (PAC+Clay) and 384t km $^{-2}$ yellow clay for treatment of *Alexandrium* bloom across historical scenarios. The yellow clay concentrations reflect the amount used during a red tide event in Korea in the field [63], and 0.1g L^{-1} reflects the concentrations used from our lab experiments.

Area of Bloom (Ha)	Location	Impacts of bloom	Cell density (cell L^{-1})	Cost estimates		Reference
				KPAC (140.8kg ha ^{1})	Yellow loess (3840kg ha ^{1})	
86	Cristo lagoon Venezuela	Recreational Ecosystem Human health	*A. minutum* 1.3x10^{7}	$1145	$53,838	[88]
200	Crique-de-l'Angle France	Human health Commercial aquaculture and Shellfisheries	*A. catenella* 4.5x10^{6}	$2,665	$122,880	[66]
650	Hunts Bay, Kingston, Jamaica	Recreational Public health	*A. minutum* 4.6 ×10^{5}	$8,661	$399,360	[77,78]
700	Syracuse Sicily, Italy	Commercial aquaculture and Shellfisheries Public health	*A. minutum* 1.0x10^{6}	$9,327	$430,080	[75]
1500	Thau Lagoon France	Human health Commercial aquaculture and Shellfisheries	*A. catenella* 4.5x10^{6}	$19,987	$921,600	[66,67]
1900	Swan River 2019/2020 bloom	Recreational Public health	*A. minutum* 1.0x10^{7}	$25,317	$1,167,360	[29]

	Western Australia					
2400	North Lake of Tunis Tunisia	Recreational	*A. catenella* $5.5x10^4$	$31,979	$1,474,560	[79]
7500	Thau Lagoon France	Human health Commercial aquaculture and Shellfisheries	*A. catenella* $3.5x10^5$	$99,934	$4,608,000	[89]
